# Engineering a medical device for intranasal immunisation: ensuring targeted delivery and formulation stability

**DOI:** 10.3389/fddev.2026.1875011

**Published:** 2026-08-18

**Authors:** Gonçalo Farias, Arnaud Bude, Nektaria Karavas, Christelle Rossignol, Isabelle Lantier, Julie Suman, Nicolas Aubrey, Isabelle Dimier-Poisson, Mathieu Epardaud

**Affiliations:** 1 Aptar Pharma, Le Vaudreuil, France; 2 Aptar Pharma, Congers, NY, United States; 3 INRAE, Université de Tours, ISP, Nouzilly, France; 4 Lovaltech, Tours, France

**Keywords:** mucosal vaccination, nasal vaccination, nasal-associeted lymphoid tissue (NALT), intranasal delivery, vaccine, aerosolisation stability, targeted aerosol delivery

## Abstract

**Introduction:**

The efficacy of nasal vaccination critically depends on the delivery method, which must ensure precise and reproducible deposition of the vaccine within the nasal cavity to effectively stimulate mucosal immune structures such as the nasal\x{2011}associated lymphoid tissue (NALT). Transitioning from pre\x{2011}clinical models to human applications is challenging because of anatomical and physiological differences in nasal structure and airflow dynamics.

**Methods:**

We developed a nasal\x{2011}spray device specifically designed for human nasal immunization. The device delivers a broad, homogeneous spray that is confined to the nasal cavity, thereby avoiding the lower respiratory tract and minimizing the risk of pulmonary exposure. It was engineered to be compatible with a wide range of vaccine formulations, preserving their physicochemical properties, stability, and delivery efficacy. To overcome the limitations of conventional rodent models, the device was validated using (i) in\x{2011}vitro human nasal cavity replicas (nasal casts) and (ii) an in\x{2011}vivo rabbit model, which is more appropriate for regulatory and translational studies.

**Results:**

The spray system achieved consistent, homogeneous deposition throughout the nasal cavity without spill\x{2011}over into the lower airways. Compatibility tests confirmed that the device does not alter the physicochemical characteristics or stability of a representative complex vaccine formulation. Validation in nasal casts and the rabbit model demonstrated reproducible delivery performance and supported the device\x{2019}s suitability for human use.

**Discussion:**

The presented transitory delivery platform offers a scalable, safe, and formulation\x{2011}compatible solution for intranasal vaccine administration. By ensuring targeted deposition and preserving vaccine integrity, the device reinforces the potential of mucosal immunization strategies across diverse indications and facilitates the translation of nasal vaccines from pre\x{2011}clinical research to clinical application.

## 1 Introduction

Nasal vaccination represents a promising alternative to traditional systemic-restricted immunisation, particularly for respiratory pathogens such as SARS-CoV-2. Unlike these classical vaccination routes, which induce primarily circulating IgG responses, nasal vaccination is not restricted to the systemic immune system but also targets the mucosal immune system, leveraging the unique properties of mucosal immunity (Davis, 2001; Yusuf and Kett, 2017; Lobaina Mato, 2019). This includes the production of secretory IgA antibodies at the site of pathogen entry and the activation of interconnected mucosal-associated lymphoid tissues, thereby enabling systemic immunity and localised immune protection to reduce viral transmission at its source.

A broad-spectrum nasal COVID-19 vaccine based on a multivalent antigenic strategy, selecting two critical viral proteins to induce efficient immunity and maintain efficacy across emerging variants has been recently developed (Lakhrif et al., 2025). To ensure targeted delivery and safety, a novel formulation was developed with an original mucoadhesive, biocompatible nanocarrier (muco-excipient), without adjuvant, allowing transmucosal access of the antigen to the Nasal-Associated Lymphoid Tissue (NALT) for the installation of a strong immune response in the nasal mucosa while minimising systemic side effects.

A critical and often underestimated aspect of nasal vaccination is the delivery method. Transitioning from preclinical models to human application poses significant challenges, primarily due to anatomical and physiological differences in nasal structure and respiratory dynamics. In classical rodent preclinical animal models such as mice and hamsters, intranasal instillation with a micropipette result in deposition in both the nasal cavity and upper lungs, a phenomenon aggravated by parameters such as the volume delivered and the anaesthesia, inadvertently enhancing immune responses beyond the simple nasal immune response through pulmonary exposure (Southam et al., 2002). In humans, however, a strictly nasal delivery could be prioritised in the objective of avoiding the risk of potential adverse effects, such as uncontrolled chronic pulmonary inflammation or cytokine storm. Nonetheless, optimal induction of the NALT for efficient vaccination in humans remains a challenge due to: the low residence time in the nasal cavity linked to the mucociliary clearance, the limited knowledge around adjuvants for intranasal delivery, the presence of enzymatic activity in the nasal cavity, and the compatibility with the device to deliver a biologically active product to the NALT (Lobaina Mato, 2019). Some examples of early clinical failures observed with intranasal spray formulations underscore the importance of a well-adapted delivery system (device and formulation) (Madhavan et al., 2022).

To address this, a nasal spray drug product was specifically developed for immunisation (Laube et al., 2024). The main goal was to ensure precise deposition of the vaccine within the nasal cavity, particularly targeting the NALT, which harbours the key immune cells responsible for initiating mucosal immunity and to ensure it stays long enough in this region to induce an immune response (Debertin et al., 2003). To this aim, the spray was developed to meet the requirements of delivery restricted to the nasal cavity (without reaching the upper respiratory system) but sufficiently broad and homogeneous to be able to target the majority of the NALT. The primary aim of this study, consistent with earlier work from Dr Goshahi’s team, was to deliver the payload to immune-cell-rich zones of the nasal cavity (Wilkins et al., 2021; Li et al., 2023; Hejazi et al., 2026). To maximize feasibility, we focused on the nasal turbinates, particularly the inferior and middle turbinates, rather than the nasopharynx region, which is anatomically less accessible. Moreover, we deliberately limited deposition in the olfactory epithelium and paranasal sinuses, because material in the former raises concerns of central-nervous-system exposure, while retention in the latter may provoke sinusitis or chronic irritation (Li et al., 2023). Another main aim of this work was to confirm that the BiVax device developed by Aptar Pharma can maintain the integrity of a vaccine formulation, a prerequisite before evaluating its capacity to deposit the dose precisely in the intended nasal regions. Accordingly, the device was challenged for its ability to preserve the formulation’s quality without inducing any degradation. Finally, recognising the limitations of conventional preclinical models, the drug product was validated using *in vitro* human nasal cavity replicas (nasal casts) and the rabbit preclinical model, already approved for preclinical regulatory toxicity assay, and suited to test *in vivo* this developed human nasal vaccine dedicated delivery spray.

Thereby, this tailored nasal spray delivery system came to optimally complete our strategy, combining innovative antigen design and advanced mucoadhesive nanocarriers, laying the foundation for a safe, effective, and scalable nasal vaccine platform.

## 2 Materials and methods

### 2.1 Manufacturing of the vaccine formulation

LVT-001 Drug Substance (DS) is a heteromultimeric recombinant protein (also referred as P12-41 protein), encoding both the spike (S) protein and nucleoprotein (N) of SARS-CoV-2 (protein antigen). The vaccine fusion protein (VFP) is composed of two polypeptide chains: (1) The heavy chain (HC) consists of the Wuhan ectodomain of the S protein fused to a trimerization domain (T4 foldon), followed by a dimerization domain (fragment crystallizable [Fc] domain of a human antibody [IgG1]) and the nucleoprotein. (2) The light chain (LC) consists of the Wuhan ectodomain of the S protein fused to the trimerization domain (Lakhrif et al., 2025).

**FIGURE 1:**
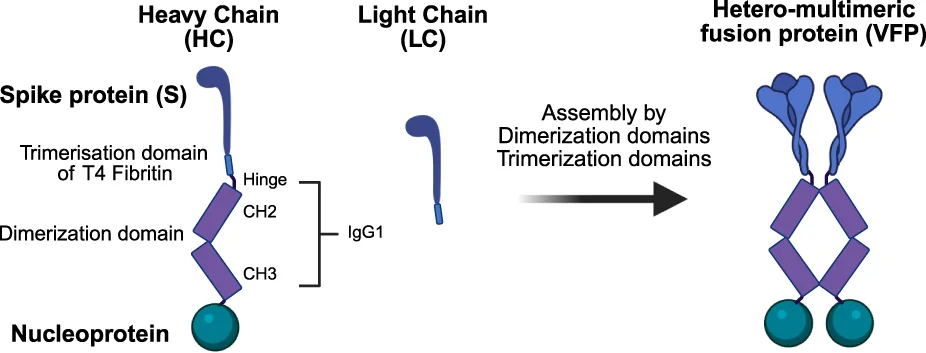
Schematic representation of the P12-41 vaccine fusion protein (VFP). The VFP is composed of two types of polypeptide chains that assemble into a functional immunogen: the heavy chain (HC) carrying the Nucleoprotein (N) fused to the SARS-CoV-2 Spike protein (S) ectodomain and the light chain (LC) displaying a Spike protein (S) ectodomain. Two HC molecules dimerize through their hinge region and the CH2–CH3 Fc–like domains. Two LC associate with the Spike ectodomain of an HC to generate a trimeric Spike architecture thanks to the T4 bacteriophage foldon trimerization motif. The overall VFP therefore comprise 2HC + 4LC.

LVT-001 HCs dimerize via the dimerization domain (hinge-CH2-CH3 of human IgG1). The LC in LVT-001 vaccine allows the trimerization of the S protein, as several light chains can self-assemble via the trimerization domain, with the HC dimer. The trimerization of the S protein in COVID-19 vaccines is a critical aspect of vaccine development as it has been shown to enhance vaccine efficacy and stability (Wrapp et al., 2020). Thereby, the VFP is composed of 6 S proteins and 2 N proteins. The structure of VFP is shown diagrammatically in Figure 1.

Antigen preparation (LVT-001 protein, DS), formulation and quality control was performed by the Biotech CDMO GTP Bioways (Olon France). The Protein identity was measured by SDS-PAGE in reduced and non-reduced conditions and purity was measured by Size-exclusion HPLC (SE-HPLC). SE-HPLC allows to determine DS purity and degree of protein aggregation for characterization of batches. The pilot batch used in this study was reported with a 94% purity (% monomer vs. aggregates/oligomers).

**FIGURE 2:**
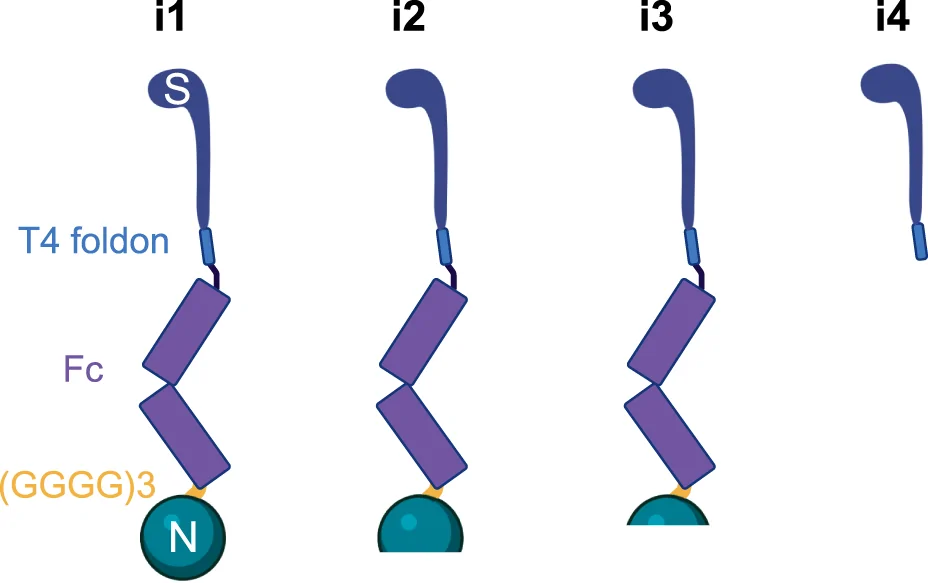
Schematic representation of P12-41 isoforms in LVT-001 drug substance (DS). Because of the inherent fragility of the nucleoprotein (N), the DS consists of a mixture of 4 isoforms representing different cleaved form of the N protein. Isoform 1 (i1), Isoform 2 (i2), Isoform 3 (i3) and Isoform 4/4′ (i4/4′), from the highest to the lowest molecular weight represent, respectively, HC molecules exposing the least-cleaved to the most-cleaved forms of the N protein.

LVT-001 DS is manufactured in recombinant Chinese Hamster Ovary (CHO) cell cultures, using standard technologies for mammalian cell growth and following several chromatographic protein purification steps. The SARS-CoV2 nucleoprotein represents a challenge in terms of production, and the result is the production of different “isoforms” resulting from the presence of different truncated forms of this protein. Thus, the LVT-001 DS is composed of a mixture of four different main isoforms of the P12-41 vaccine fusion protein, formulated in a liquid formulation buffer (phosphate-buffered saline [PBS]). The first isoform (i1) is the full-length HC composed of the S protein fused to the trimerization domain (T4 foldon), the dimerization domain (human IgG1 Fc) and the N protein, as described above. Isoforms i2 and i3 were identified by peptide mapping (data not shown) as truncated HCs, which exhibit a Cter truncation of the N protein. The i2 is truncated in the dimerization domain of the N protein (∼aa 293/419), which results in 30% loss of the N sequence. The i3 is truncated in the RNA Binding Domain of the N protein (∼aa 68/419), which results in 85% loss of the N sequence. Isoform i4 corresponds to the P12-41LC monomer. This latter isoform was further identified by LC-MS/MS as differentially glycosylated, resulting in the additional glycosylated form called i4’ (data not shown). A schematic representation of each isoform is represented in Figure 2.

The LVT-001 formulation, or Drug Product (DP), is designed to prevent SARS-CoV-2 infection by trans-mucosal vaccination via the intranasal route (IN). The DP is composed of LVT-001 DS combined with a biocompatible mucosal nanocarrier, the muco-excipient (LVT-ME) with a ratio of 3:1 (nanocarriers: antigen) within a PBS formulation buffer. This mucoadhesive delivery systems allows vaccine antigens to cross the nasal mucosa and gradually deliver antigens to immune cells, for an efficient induction of both systemic and mucosal immune response against SARS-CoV-2 variants. The LVT-ME, provided by the Lovaltech company (Lovaltech, Tours), is an original cationic nanocarrier composed of positively charged polymeric sugar matrix (maltodextrin) round particles with an average diameter of 35–70 nm. The batch of DP used in this study was stored at −20 °C and corresponded to the technical/pilot batch of the LVT-001 project.

**FIGURE 3:**
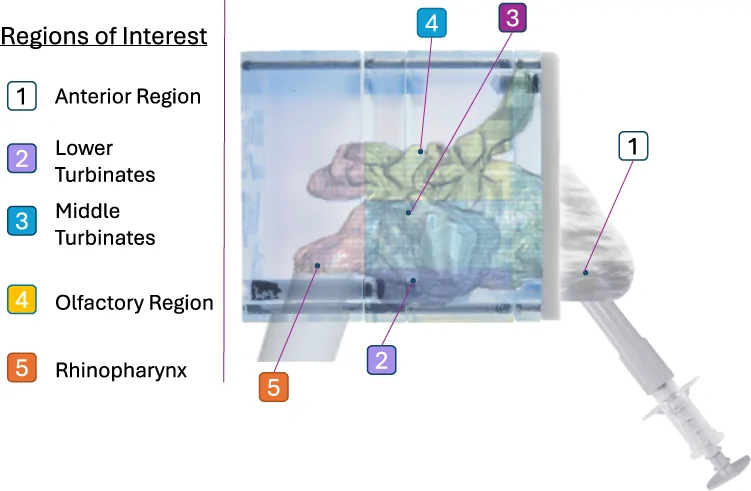
Schematic representation of the different regions of interest that were evaluated with Aeronose™ and BiVax 200 µL intranasal atomizer. (1) anterior region (nose and nasal valve) in white, (2) lower turbinates in purple, (3) middle turbinates in blue, (4) olfactory region in yellow and (5) rhinopharynx in orange.

The DP was then aerosolised with Aptar Pharma BiVax 200 µL intranasal atomiser (Aptar Pharma, Le Vaudreuil, France), as shown in Figure 3. For each experiment, a new BiVax was loaded with approximately 268 ± 7 mg of DP by removing the white actuator, piercing a rubber-topped vial with 500 µL with the syringe needle and pulling the piston with the vial upside down, ensuring no air bubbles within the device. The actuator was then reattached over the needle, and the device was actuated for each experiment detailed below.

Aside from the in-use stability studies (Table 1; Figure 5) where 0.1 g/L and 0.6 g/L concentrations were used, all experiments were performed with the DP at a concentration of 0.6 g/L. Thus, this concentration was used for the mouse pharmacokinetic study (20 μL per mouse) and the rabbit deposition study (200 μL per rabbit). It corresponds to the DP concentration employed in all previous pre-clinical experiments (20 μL per mouse) (Lakhrif et al., 2025), in the regulatory toxicity study in rabbits (200 μL per rabbit) carried out by C. Ris Pharma (from which the rabbits used in the present manuscript were obtained) and in the ongoing clinical trial (Higher dose, 200 μL per volunteer) (ClinicalTrials.gov ID NCT06821126).

**TABLE 1:** Summary table of the in-use stability study presenting average ± 1 standard deviation. Protein concentration with BCA assay and particle size with DLS were tested in triplicate before and after nebulization (at t0 after exposing the formulation to the device for 4 h and 16 h).

Test	Before aerosolization		After aerosolization					
			t0		t4 h		t16 h	
Concentration (g/L)	0.1	0.6	0.1	0.6	0.1	0.6	0.1	0.6
BCA concentration (g/L)	0.10 ± 0.01	0.60 ± 0.01	0.10 ± 0.00	0.60 ± 0.00	0.08 ± 0.01	0.59 ± 0.02	0.07 ± 0.01	0.50 ± 0.03
Z-average (nm)	-	50 ± 1	-	55 ± 1	-	52 ± 1	-	56 ± 1
PDI	-	0.30 ± 0.04	-	0.38 ± 0.03	-	0.37 ± 0.02	-	0.41 ± 0.03
ELISA Anti-S relative potency EC50% (%)	-	103 ± 3.5	-	115 ± 6.4	-	110 ± 8.5	-	84 ± 0.6
ELISA Anti-N relative potency EC50% (%)	-	104 ± 0.7	-	122 ± 0.7	-	125 ± 13.6	-	71 ± 2.1

### 2.2 Spray characterisation

Droplet size distribution was measured using a Spraytec® (Malvern Panalytical, Worcestershire, United Kingdom) equipped with a 300-mm lens. The nasal spray was actuated at a distance of 4 cm from the laser using a Vereo® Actuator NSx (Proveris Scientific, Hudson, United States) at a velocity of 50 mm/s, representative of user application, until a force of 6 kg was achieved. Measurements were taken at a frequency of 500 Hz for 0.6 s. The average droplet sizes at 10%, 50%, and 90% of the cumulative volume undersize (Dv10, Dv50, and Dv90, respectively) during the fully developed phase (0.04–0.1 s) of the spray were analysed.

Spray pattern and plume geometry were determined using a SprayVIEW® (Proveris Scientific, Hudson, United States) at a distance of 5 cm from the laser, with the same actuation machine and parameters used for droplet size measurements. Measurements were performed at a frequency of 100 Hz with a lens aperture of 4, capturing 100 images. The maximum (Dmax) and minimum (Dmin) diameters of the spray pattern and their ratio (ovality) were calculated based on the entire spray. The plume angle was assessed from a single frame during the fully developed phase. Five devices were tested in duplicate for all spray characterization testing.

### 2.3 Stability of the vaccine formulation post-spray

To evaluate the in-use stability of the DP, samples were analysed at multiple time points: prior to aerosolisation (baseline), immediately after aerosolisation, and following storage within the aerosolisation device at room temperature (20 °C ± 5 °C) for 4 h and 16 h (analysis after aerosolisation). At each time point, triplicate samples were subjected to a panel of analytical techniques to assess structural integrity, concentration, and biological activity. These included the Bicinchoninic Acid (BCA) assay for protein quantification, Native Polyacrylamide Gel Electrophoresis (Native-PAGE) for structural assessment, Dynamic Light Scattering (DLS) for particle size distribution, and Enzyme-Linked Immunosorbent Assay (ELISA) for potency evaluation. Two different concentration levels of LVT-001 DP (0.1 and 0.6 g/L) were tested to cover the clinical trial concentration range. Indeed, the Phase I clinical studies were carried out by administering 200 µL per volunteer using the BiVax 200 device, comparing three formulation concentrations, the lowest at 0.1 g/L and the highest at 0.6 g/L (ClinicalTrials.gov ID NCT06821126).

#### 2.3.1 Protein quantification using the BCA assay

Protein concentration was determined using the bicinchoninic acid (BCA) assay. This method is based on the reduction of Cu^2+^ to Cu^+^ by proteins in an alkaline medium, followed by the formation of a purple-colored complex between Cu^+^ and BCA, which is measured at 562 nm. The reaction is particularly sensitive to cysteine, cystine, tryptophan, and tyrosine residues, though the peptide backbone also contributes to signal generation, minimising variability between different protein types.

Bovine serum albumin (BSA) standards and samples were loaded in duplicate into a 96-well plate, followed by nine 2-fold serial dilutions in 1X PBS (137 mM NaCl, 2.7 mM KCl, 4.3 mM Na_2_HPO_4_, 1.4 mM KH_2_PO_4_, pH 7.4). The BCA working reagent was prepared by mixing 400 µL of Reagent B with 20 mL of Reagent A. A volume of 225 µL of the BCA reagent was added to each well. The plate was incubated for 30 min at 37 °C without shaking, followed by an additional 15 min at room temperature. Absorbance was read at 562 nm, and protein concentrations were determined using a standard curve generated with BSA.

#### 2.3.2 Native-PAGE analysis of drug substance/muco-excipient complex

The Native-PAGE method was used to monitor the complexation between the DS, P12-41 protein, and the muco-excipient (ME), which results in the formation of the final DP. In this assay, complexation is inferred from the absence of protein migration into the gel. Since ME is positively charged and too large to migrate in native gels, its complex with P12-41 prevents the protein from entering the gel during electrophoresis.

Protein complexation was assessed using the NativePAGE™ Novex® Bis-Tris Gel system (3%–12% gradient) under non-denaturing conditions, based on the Blue Native-PAGE (BN-PAGE) method described by Schägger and von Jagow (Schägger and von Jagow, 1991). Coomassie G-250 in the cathode buffer provides proteins with a net negative charge without denaturation, enabling separation while maintaining native conformations.

Samples were prepared by mixing 26 µL of the test sample with 10 µL of 4X Native-PAGE sample buffer and 4 µL of ultrapure water. No heating step was applied. A total of 13.1 µL of the prepared sample was loaded per well. Electrophoresis was performed at 150 V with a maximum current of 80 mA per gel for 100 min in 1X anode and 1X cathode running buffers. The electrophoresis unit was kept under an ice blanket to prevent overheating.

Following electrophoresis, gels were washed three times in water for 5 min, stained with Bio-Rad BioSafe™ Coomassie stain for 20 min, then destained with three washes of 20 min and one final wash of 3 h in water. Gels were scanned to visualise protein bands.

#### 2.3.3 Dynamic light scattering (DLS)

DLS was employed to determine the particle size distribution of the DP. The technique is based on the analysis of intensity fluctuations in scattered light caused by the Brownian motion of particles when illuminated with a monochromatic, coherent light source (laser). These fluctuations provide information on the diffusion coefficients, which are used to calculate particle size using the Stokes–Einstein equation. DLS enables the analysis of particles within a size range of 0.3–10,000 nm. Measurements were performed using a Zetasizer instrument (Malvern Panalytical, Worcestershire, United Kingdom) with semi-micro PMMA cuvettes. For measurement, DP samples are diluted in 15 mM NaCl at 10 g/L. All measurements were conducted in triplicate (n = 3).

#### 2.3.4 Potency assay by indirect competitive ELISA

The potency of the LVT was evaluated using two indirect competitive ELISAs targeting the Spike (S) protein and Nucleoprotein (N) domains of the fusion molecule. 96-well plates were coated with the LVT reference substance at 250 ng/mL in PBS and incubated for 75 min at 20 °C. After washing, serial dilutions of the test samples were pre-incubated with either anti-N protein polyclonal rabbit antibody (0.945 μg/mL; OriGene, TA890230) or anti-Spike protein polyclonal rabbit antibody (16 ng/mL; Rockland, 600–401-MS9) in dilution buffer (PBS, 0.1% Tween 20, 1% BSA). Sample concentration ranges were 4.69–3,000 ng/mL (N ELISA) and 1.25–30000 ng/mL (S ELISA). Antigen-antibody mixtures (1:1, v/v) were incubated for 90 min at 20 °C before transfer to the coated plates for a further 60 min incubation. Following washes, plates were incubated with HRP-conjugated goat anti-rabbit IgG (1:7500) for 30 min, washed again, and developed using TMB substrate for 20 min at 20 °C. The reaction was stopped with 1 M HCl, and absorbance was measured at 450 nm using a BIOTEK Elx808 microplate reader.

### 2.4 Nasal cast regional deposition

*In vitro* deposition of BiVax liquid nasal sprays was characterised in an adult male nasal cast (Aeronose™, courtesy of Aptar/DTF/Univ. Of Tours) with chemical quantification (Figure 3). The nasal cast model was designed from computed tomography (CT) images of a plastinated head model (Durand et al., 2011) and was previously validated as a predictive model for nasal aerosol deposition (Williams et al., 2021).

An initial evaluation of the best administration angle and insertion depth with BiVax was performed with an aqueous 75 mg/mL fluorescein sodium solution. One actuation per nostril was manually actuated into the nasal cast using a combination of different actuation positions, which were accurately maintained with a jig: insertion depths of 2, 9, and 15 mm; delivery angles (horizontal plane) of 30°, 45°, and 50°; and a fixed vertical angle of 5° from the center wall, slightly tilted towards the septum (Figure 3). During actuation, no flow and 15 L/min were applied. The nasal cast was also tested in different positions: upright (Up), 45° (45), and supine (Sup), with and without airflow (15 L/min).

After confirming the optimal administration parameters, the DP was gently mixed with the original fluorescein sodium solution (75 mg/mL) to achieve an homogeneous solution with a final concentration of 13.5 mg/mL for nasal cast quantification. The aim of this evaluation was to verify that the nasal cast deposition tendencies would be consistent between the aqueous fluorescein solution and the final DP.

The different regions of interest (anterior region, olfactory region, middle turbinates, lower turbinates, nasopharynx, and filter) were rinsed with varying volumes of water, depending on the volume required to cover these regions. Samples were then quantified using a spectrophotometer (Shimadzu UV-2600i, Noisiel, France) with a calibration curve ranging from 0.1 to 10 μg/mL. Results are expressed as the percentage of the administered dose recovered in each region of interest, following normalization to the total recovered dose. Mass balance for each replicate was calculated as the ratio of the total recovered mass to the theoretical mass, the latter derived from the known formulation concentration and delivered spray mass. All mass balances were within an 87%–108% range for the 75 mg/mL aqueous fluorescein solutions and between 82% and 87% for the combination of DP with fluorescein. The comparatively lower recovery observed for the DP formulation may be attributed to differences in formulation relative to the calibration standards.

### 2.5 Preclinical study

#### 2.5.1 *In Vivo* nasal kinetic distribution of the formulation in mice

Six-week-old female BALB/c mice (CER Janvier, Le Genest-Saint-Isle, France) were housed under specific pathogen-free (SPF) conditions in a BSL-2/3 animal facility at the Experimental Infectiology Platform (PFIE, INRAE, UE-1277, Val de Loire Research Center, Nouzilly, France). Mice received intranasal immunisation with the DP mixed with fluorescein. To this aim, 2 mL of DP were gently mixed with 10 mg of fluorescein to achieve a homogeneous solution with a final concentration of 5 mg/mL. A total volume of 20 µL (10 µL per nostril) of the treatment solution (containing 10 mg fluorescein in 2 mL) was administered using a micropipette under light anaesthesia. Mice were euthanised, by isoflurane overdose followed by cervical dislocation, at 15, 30, and 45 min post-administration for kinetic analysis of nasal deposition. The heads were collected and fixed in 4% paraformaldehyde. Fluorescence imaging was performed using the IVIS Spectrum Imaging System (PerkinElmer, Waltham, MA, United States) to evaluate both fluorescein distribution and intrinsic tissue autofluorescence. Quantification of fluorescence was achieved by subtracting background autofluorescence from the total signal. Data were expressed as average radiant efficiency ([p/s/cm^2^/sr]/[µW/cm^2^]). The fluorescence detection threshold was defined as the mean signal plus two standard deviations of the corresponding signal measured in untreated control tissues.

The purpose of this kinetic study was solely to identify the optimal time window for detecting the *in vivo* nasal distribution of the formulation. Consequently, only a single animal per condition was used, as prior observations by other collaborators had already demonstrated comparable results across replicates (data not shown). Nevertheless, the data were weighted by averaging the fluorescent-formulation distribution across each half of the nasal cavity, thereby accounting for the DP being distributed equally between the two nostrils; consequently, each half-head could be treated as an independent biological replicate corresponding to the administration in a single nostril.

#### 2.5.2 *In Vivo* nasal distribution of the formulation in rabbits nasal cavity using BiVax 200 µL intranasal atomiser

Rabbit *in vivo* experiments were conducted at the experimental facility of C. RIS Pharma (Saint-Malo, France) to evaluate the distribution of the vaccine formulation within the nasal cavity and turbinates following administration with the BiVax. New Zealand White (NZW) albino rabbits were selected as the animal model, as this species is widely accepted by regulatory agencies for preclinical vaccine testing and would further be used for regulatory toxicity studies. Healthy male NZW rabbits (14–24 weeks old) were procured from Charles River Laboratories (France).

Animals were acclimated for at least 5 days in the conventional animal care unit prior to the start of the study. The animal care unit at C. RIS is accredited by the French Ministries of Agriculture and Research (authorisation No. C35-288-01). All experimental procedures were conducted in accordance with ethical guidelines for animal experimentation. The protocol was reviewed and approved by the local Animal Care and Ethics Committee (CEGCP: “Comité d’Éthique du Groupe Cellis Pharma”) on 2 December 2022.

**FIGURE 4:**
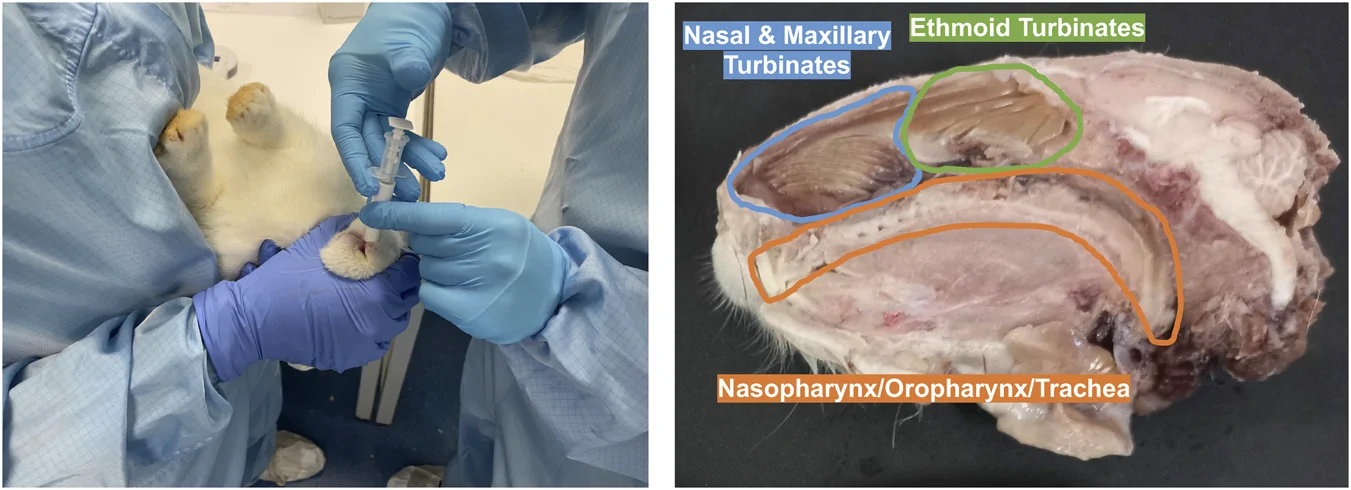
Intranasal administration of the DP using BiVax 200 µL intranasal atomizer in a rabbit (Left) intranasal administration in rabbit of the DP using the BiVax 200 µL. The rabbit was held in an inverted position during the administration and maintained in this position for 1 minute following instillation (similar method used with micropipette). (Right) Longitudinal section of the rabbit head with overlayed schematic localization of the different regions of interest in the rabbit nasal cavity: (1) nasal and maxillary turbinates in blue, (2) ethmoid turbinates in green, nasopharynx-oropharynx-trachea in orange.

Intranasal (IN) administration of the DP mixed with fluorescein, to achieve a homogeneous solution with a final concentration of 5 mg/mL, was performed in one animal using the BiVax 200 µL device, and as a control, with a micro pipette in another animal. Each rabbit received 100 μL per nostril. Following administration, animals were manually restrained for 1 minute to ensure proper distribution (Figure 4 left). Fifteen minutes post-administration, rabbits were anesthetized and euthanized for analysis of nasal deposition within the global nasal cavity: nasal and maxillary turbinates, ethmoid turbinates, nasopharynx, oropharynx and trachea (Figure 4 right). Animals were anesthetized under isoflurane then euthanized (after intracardiac sampling) by a lethal intracardiac injection of Dolethal (1 mL/kg) and carotid arteries were cut for final exsanguination.

The heads were removed, and the tissues were fixed in 4% paraformaldehyde. Optical imaging was performed using the IVIS Spectrum Imaging System (PerkinElmer, Waltham, United States) to assess fluorescein distribution and tissue autofluorescence. Fluorescence quantification was conducted by subtracting tissue autofluorescence from the fluorescein signal. The results were expressed as average radiant efficiency ([p/s/cm^2^/sr]/[μW/cm^2^]). The detection threshold for fluorescence was defined as the mean signal plus two standard deviations of the corresponding signal from control (untreated) rabbit tissues.

These studies on the nasal localization of the vaccine formulation in rabbits were carried out as part of regulatory toxicology testing in collaboration with C-Ris Pharma. Because of the limited number of animals available, stemming from regulatory requirements that the experiments be conducted within the framework of toxicology studies, only one animal per treatment group (Bivax, micropipette, control) was employed. Nevertheless, the data were weighted by averaging the fluorescent-formulation distribution across each half of the nasal cavity, thereby accounting for the Drug Product (DP) being distributed equally between the two nostrils; consequently, each half-head could be treated as an independent biological replicate corresponding to the administration in a single nostril.

### 2.6 Statistical analysis

For the evaluation of vaccine formulation stability post-spray, statistical comparisons between groups were performed using one-way analysis of variance (ANOVA), followed by unpaired t-tests where appropriate.

A multivariate analysis was conducted to assess the impact of various factors (administration angle, insertion depth, model position, and airflow) on deposition distribution. Data for the filter, nasopharynx, and olfactory region were excluded from the multivariate analysis due to negligible deposition in these areas. Unpaired t-tests were performed to evaluate the significance of each coefficient associated with the variables in the multivariate model.

To compare the fluorescein solution with the final DP, unpaired t-tests were performed for each region of interest.

All statistical analysis were performed using SOS Stat software (version 3.5.0.6, Doussard, France). Probability values of <0.05 were considered as statistically significant.

## 3 Results

### 3.1 Spray characterization

The average Dv10, Dv50, and Dv90 values, along with their standard deviations, were 18 ± 1 μm, 32 ± 1 μm, and 57 ± 3 μm, respectively. The average fraction of droplets below 10 µm and the spray angle, with their standard deviations, were 1.5% ± 0.6% and 58° ± 9°, respectively.

### 3.2 Stability of the vaccine formulation post-spray

**FIGURE 5:**
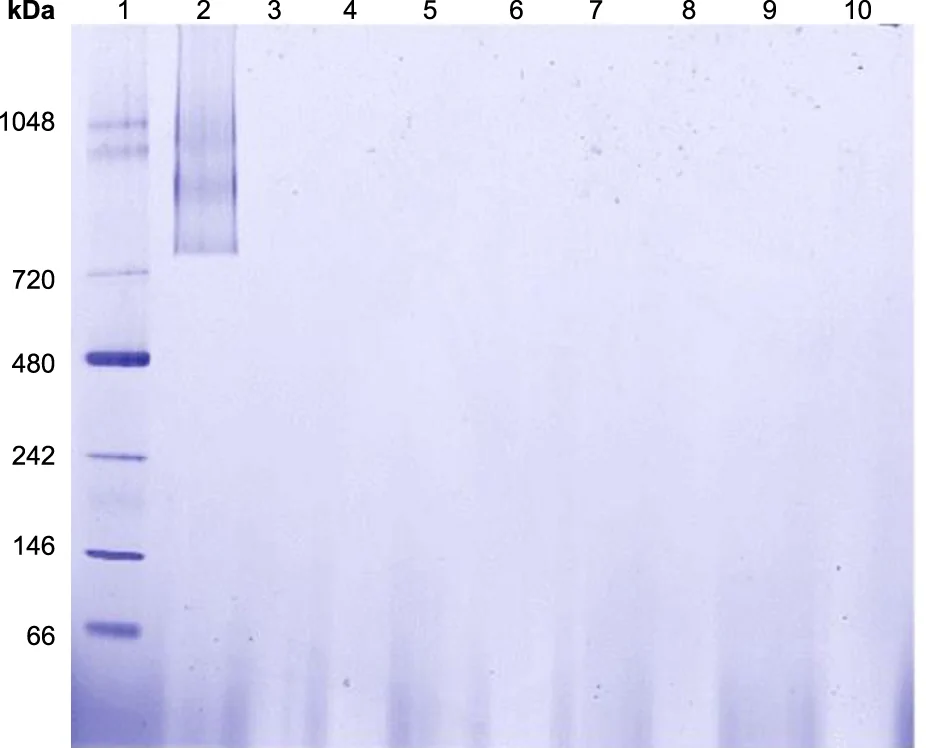
Representative Native-PAGE analysis of the DP before and after aerosolization. Lane 1: molecular weights protein ladder (66, 146, 242, 480, 720 and 1048 kDa). Lane 2: DS control sample (not formulated with the muco-excipient). Lane 3 and 4: DP (composed of DS formulated with the muco-excipient) before aerosolization at 0.1 g/L (lane 3) and at 0.6 g/L (Lane 4). Lane 5 and 6: DP after aerosolization (0 h) at 0.1 g/L (lane 5) and at 0.6 g/L (Lane 6). Lane 7 and 8: DP 4 h post-aerosolization at 0.1 g/L (lane 7) and at 0.6 g/L (Lane 8). Lane 9 and 10: DP 16 h post-aerosolization at 0.1 g/L (lane 9) and at 0.6 g/L (Lane 10).

The in-use stability of the DP results after aerosolisation and exposure to the device for 4 and 16 h is presented in Table 1 and Figure 5. Protein concentration, as assessed by the BCA assay, remained stable after aerosolisation for both samples (p > 0.05). However, a slight reduction was observed at both T4 and T16 for the 0.1 g/L dose (p < 0.01), and at T16 for the 0.6 g/L dose (p < 0.01).

As visible in Figure 5, results for Native PAGE showed an absence of free protein in the samples before and after aerosolisation (no signal), demonstrating that LVT-001 DS remains complexed with LVT-ME even after aerosolisation and exposure to the device.

Regarding the particle size evaluated by DLS, the method was suitable only for the samples at a concentration of 0.6 g/L (Table 1). For the 0.6 g/L dose, a slight increase (p < 0.05) in Z-average and PDI after aerosolization was observed. However, no significant difference was observed after incubation of the DP in the device for 16 h.

To investigate LVT-001 protein functionality we developed a dedicated potency assay based on an indirect competitive ELISA assay. This test is composed of two enzyme-linked immunosorbent assays (ELISA) targeting the two main domains of the fusion protein: anti-S and anti-N. The indirect competitive ELISA is a variant of the indirect ELISA, which allows quantifying the target antigen by inhibiting the binding of the antibodies to the same antigen immobilised on the wells of an ELISA microtiter plate. For dose 0.1 g/L, the protein concentration was on the limit of detection, hence results are not presented herein. For dose 0.6 g/L, as it was observed with BCA results, there is no impact of the aerosolisation, however there is a slight decrease in potency after exposure to the device for 16 h (Table 1).

### 3.3 Nasal cast regional deposition

**FIGURE 6:**
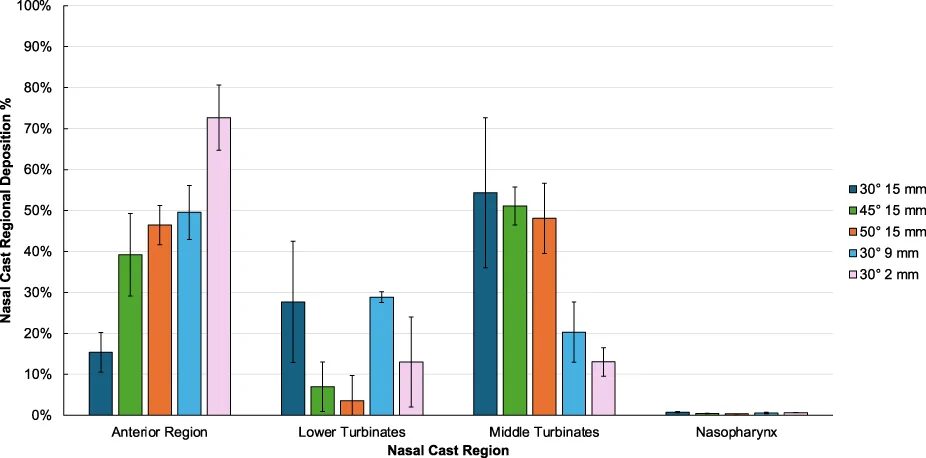
Regional deposition of a fluorescein solution in a nasal cast model assessed for different device insertion and administration angles. Administration of the fluorescein solution were all performed in an upright position with no-airflow conditions. Different device insertion depths were tested while maintaining the same insertion angle (30°): 2, 9, and 15 mm; shown in different shades of blue. Different administration angles were tested while maintaining the same insertion depth (15 mm): 30°, 45°, and 50°; shown in distinct colours. Each condition was tested in triplicate. Histogram bars represent the mean percentage deposition ±1 standard deviation across the different regions of interest. Statistical analysis was performed using a multivariate approach, as detailed in Table 2.

**FIGURE 7:**
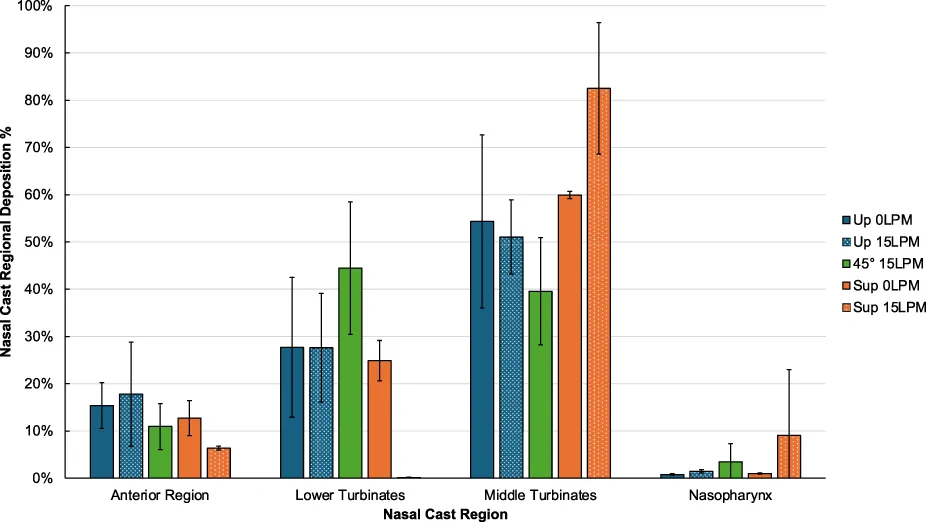
Regional deposition of a fluorescein solution in a nasal cast assessed across different nasal cast orientations and airflow conditions. All experiments were conducted using a 30° administration angle and 15 mm insertion depth. Each condition was tested in triplicate. Administrations of the fluorescein solution were tested with different nasal cast orientations: upright, 45° inclination, and supine; shown in distinct colours. All these conditions were tested with and without airflow; shown in different bar patterns. Histogram bars represent the mean percentage deposition ±1 standard deviation across defined nasal regions. Statistical analysis was performed using a multivariate approach, as detailed in Table 2.

The regional deposition of a fluorescein aqueous solution with BiVax, comparing different administration angles and insertion depths in the upright position and without airflow, is presented in Figure 6. The optimised administration position (30° and 15 mm insertion depth) was then used to evaluate different nasal cast positions and the impact of airflow, as shown in Figure 7. In both figures, the deposition in the olfactory region and filter is not presented as it is negligible (less than 2% and 0%, respectively). A multivariate analysis was conducted, and the results are presented in Table 2. This analysis revealed that airflow did not significantly affect regional deposition. In contrast, the administration angle, insertion depth, and model position had a significant impact.

**TABLE 2:** p-value results of multivariate analysis of variance of different input parameters: angle, insertion depth, model position and airflow. A p-value less than 0.05 indicates a statistically significant difference.

Region of interest	Angle	Insertion depth	Model position	Airflow
Anterior region	<0.01	<0.01	0.04	0.33
Lower turbinates	<0.01	0.06	0.05	0.65
Middle turbinates	0.59	<0.01	0.01	0.69

At a fixed insertion depth of 15 mm, increasing the administration angle from 30° to 50° led to a marked increase in anterior region deposition—from 15% to 46%. This shift was accompanied by a substantial reduction in posterior deposition, particularly in the lower turbinates, where average deposition decreased from 28% at 30° to just 4% at 50°. In contrast, deposition in the middle turbinates remained relatively stable, ranging from 48% to 54%.

Given that the 30° angle yielded the highest deposition in the posterior region and lower turbinates, this configuration was further tested at insertion depths of 2, 9, and 15 mm. Increasing the insertion depth from 2 mm to 15 mm significantly reduced anterior deposition from 73% to 15% on average. At 15 mm, deposition was predominantly localised in the lower and middle turbinates, with average values of 28% and 54%, respectively. In contrast, the 2 mm insertion depth resulted in the lowest deposition in these regions, averaging 13% each. Regarding the posterior deposition, a significant difference of the insertion depth was only observed for the middle turbinates.

A combination of model position (upright, supine and 45°) with and without airflow configurations were tested with the optimised administration position (30° and 15 mm insertion depth) as presented in Figure 7. Deposition in the anterior region remained consistently low across all configurations, with the highest (18%) average value observed in the upright position and the lowest (6%) in the supine position. The posterior deposition ranged from 0% to 44% for the lower turbinates and 40%–83% for the middle turbinates. Nasopharyngeal deposition remained below 1% in all vertical (upright) configurations. When airflow was introduced in the 45° and supine positions, nasopharyngeal deposition increased slightly to 3% and 9%, respectively, however, these differences were not considered statistically significant.

**FIGURE 8:**
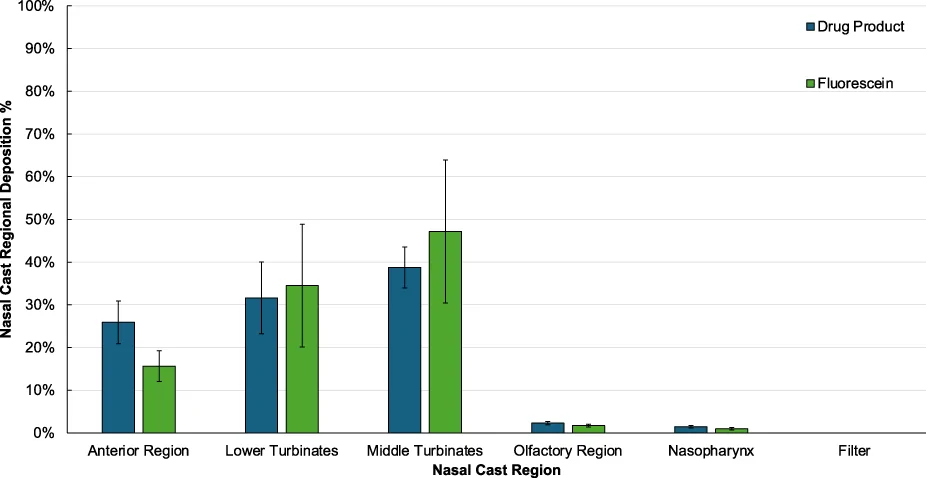
Regional deposition of a fluorescein solution versus the DP formulation in a nasal cast. All administrations were performed under upright, no-airflow conditions using a 30° angle and 15 mm insertion depth. Deposition of the fluorescein solution is shown in green and the DP formulation is shown in blue. Each condition was tested in five replicates. Histogram bars represent the mean percentage deposition ±1 standard deviation across defined nasal regions.

To confirm that the results observed with the fluorescein solution are applicable to the final DP, a comparison of regional deposition for both products was performed, as shown in Figure 8. Deposition in the olfactory region, nasopharynx, and filter remained negligible, with no statistical difference between the two products for the lower turbinates and turbinates (t-test, p > 0.05). A slight difference of approximately 10% on average was observed in the anterior region (t-test, p < 0.01).

### 3.4 Preclinical studies

**FIGURE 9:**
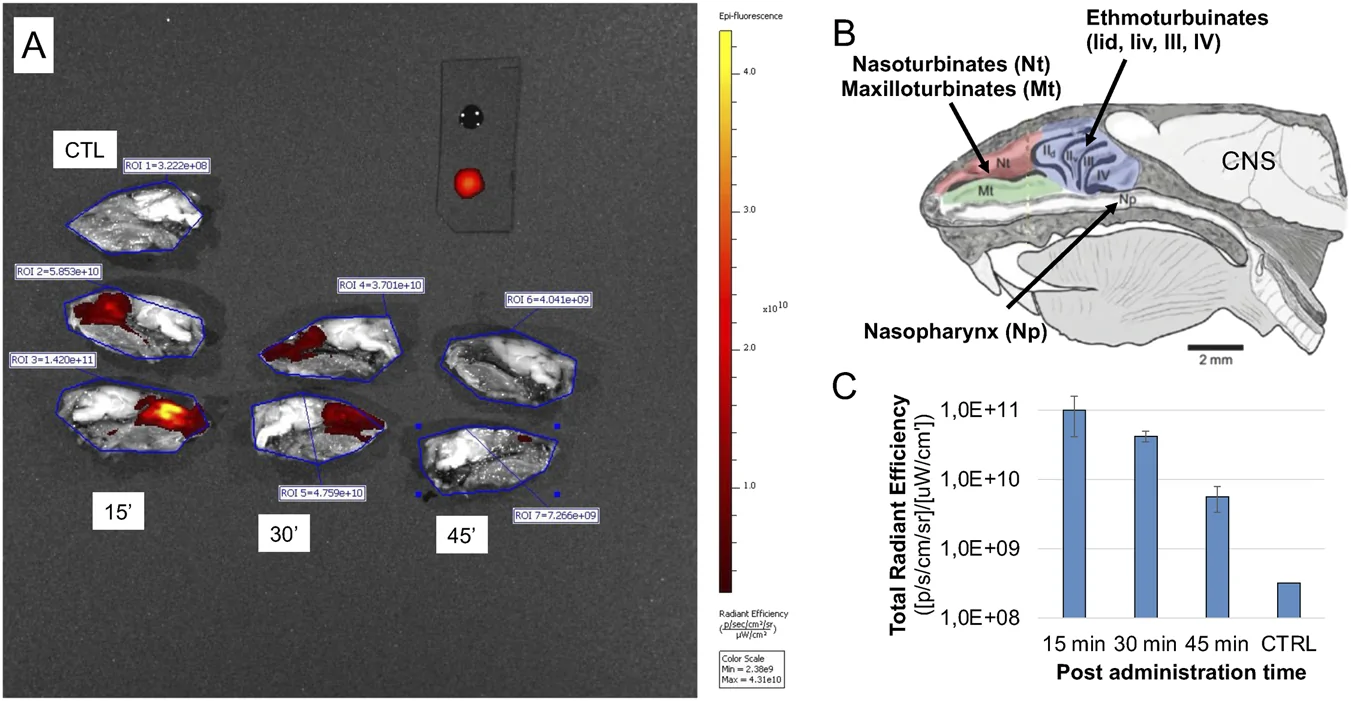
*In vivo* nasal kinetic distribution of the formulation in mice nasal cavity. **(A)** Longitudinal mice head sections visualization of the total radiant efficiency measured following fluorescent formulation administration with a micropipette, at 15, 30, and 45 min, and the control sample (no treatment) using the IVIS Spectrum Imaging System (PerkinElmer, Waltham, MA, United States). **(B)** Mouse head schematic representation of the different regions of interest in the nasal cavity with the Nasoturbinates and Maxillary turbinates (Nt and Mt, in red), the Ethmoturbinates (Iid + Iiv + III + IV, in blue) and the Nasopharynx (Np, in white). **(C)** Mean ± standard deviation of the total radiant efficiency measured from the longitudinal sections of the head at different time point post administration (see **(A)**).

To assess the detection kinetics of the formulation following intranasal administration, a pilot study was conducted using a murine model. Three mice received the fluorescein-labelled formulation via nasal instillation using a micropipette. Animals were sacrificed at three different time points: 15, 30, and 45 min post-administration. Fluorescence levels were quantified in the nasal cavity (including turbinates and nasopharynx) to evaluate formulation retention (Figure 9). The kinetic deposition analysis revealed that fluorescence intensity peaked at 15 min post-administration, with an average total radiant efficiency mean ± standard deviation of 1.0E+11 ± 0.59, followed by a progressive decrease of the measured signal over time, suggesting that this time point is optimal for evaluating the maximal intranasal distribution of the formulation (Figure 9C).

Based on the results of the kinetic deposition experiment in mice, and to further complement the results obtained with the nasal cast experiments *in vivo,* a rabbit model was employed to assess the efficiency of the BiVax device for intranasal delivery of the formulation, with a particular focus on deposition within the turbinates in the nasal cavity and the adenoid region nest to the pharynx, both regions representing abundant immune-associated tissues that may support mucosal immune responses.

**FIGURE 10:**
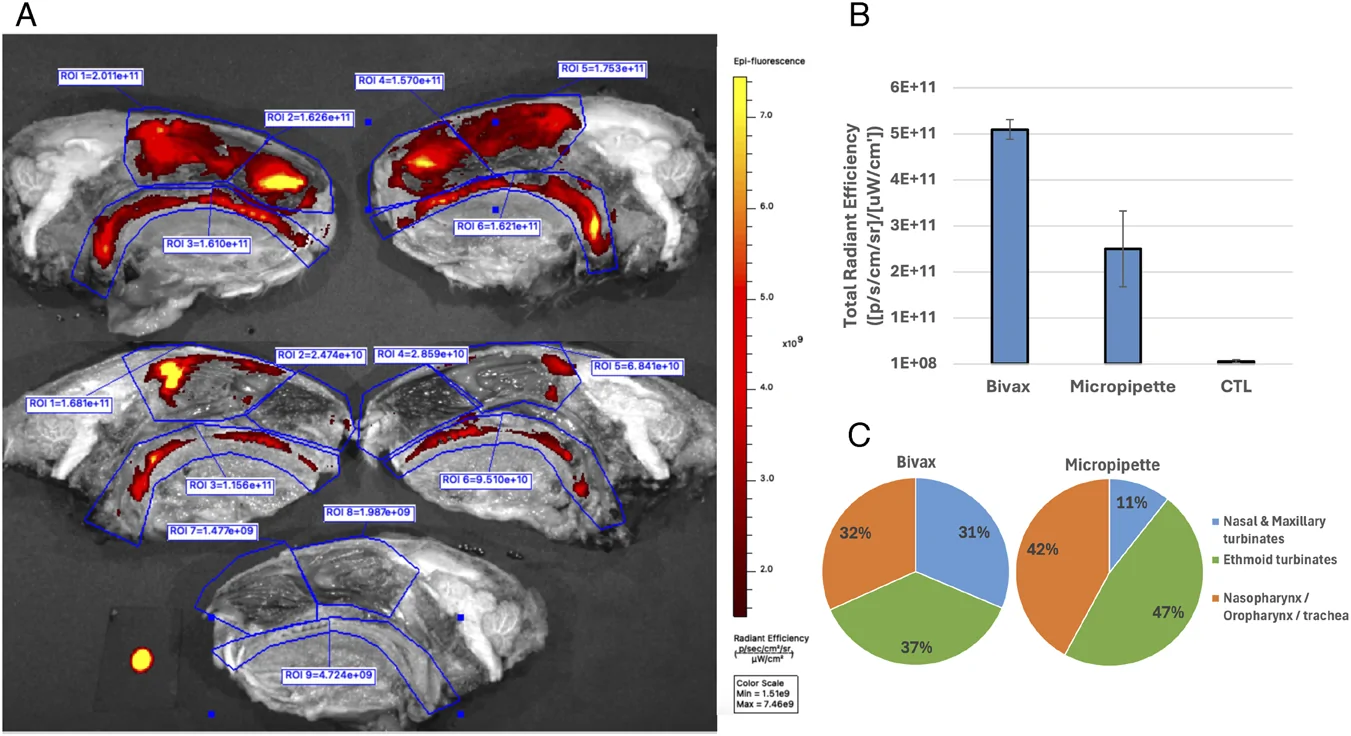
*In vivo* nasal distribution of the formulation in rabbit nasal cavity using the BiVax 200 µL intranasal atomizer and a micropipette. **(A)** Longitudinal rabbit head sections visualization of the radiant efficiency following fluorescent formulation administration using the BiVax (top left and right), micropipette (middle left and right) and control (bottom). **(B)** Mean ± standard deviation of the total radiant efficiency measured from the longitudinal sections (see **(A)**). **(C)** Percentage of the total measured radiant efficiency distribution within the different regions of the nasal cavity following BiVax and micropipette administration.

To this end, 200 μL of fluorescein-labelled formulation (100 μL per nostril) were administered to one animal using the BiVax and another animal using a micropipette to compare both method of administration. Intranasal deposition was assessed 15 min post-administration, in accordance with the kinetics observed in mice, as this time point corresponds to the peak formulation detection following delivery. Since the formulation was administered in two half-doses, one per nostril, results are represented with the mean ± standard deviation of the total radiant efficiency within the regions of interest measured on each half of the animal’s head. The total radiant efficiency within the regions of interest is calculated by the addition of the 3 regions: (1) Nasal and Maxillary turbinates, (2) Ethmoid turbinates and (3) Nasopharynx-Oropharynx-trachea area (Figure 10A).

The first observation was a total radiant efficiency mean ± standard deviation obtained following BiVax administration higher than following micropipette administration, with an average of 5.10E+11 ± 2.14 vs. 2.50E+11 ± 8,23, respectively (Figure 10B). A more detailed analysis of signal distribution throughout the nasal cavity, nasopharynx, and upper trachea reveals that administration using the BiVax device also results in a more homogeneous deposition in the nasal cavity, particularly in the nasal turbinates with a more balanced deposition between nasal and maxillary and ethmoid turbinates (Figure 10C). Indeed, for a relatively similar global deposition within the whole turbinates, i.e. 68% with the BiVax compared to 58% with the micropipette, about 31% of the formulation was detected in the nasal and maxillary turbinates when delivered with BiVax, compared to only 11% with micropipette administration deposited in this area, leading to an unbalanced distribution within the total turbinates (Figure 10C).

## 4 Discussion

This proof-of-concept study evaluated the feasibility of using a formulation targeting SARS-CoV-2 via intranasal administration of a protein incorporated in a muco-excipient, delivered using the BiVax device (Lakhrif et al., 2025).

Intranasal administration is a well-established route, currently employed for both locally acting products (e.g., Nasonex®) and systemic emergency treatments (e.g., Narcan®, Neffy®) encompassing both small molecules and biologics (e.g., Miacalcin®, Baqsimi®, Syntocinin®) (Scherließ, 2020). However, the aerosolisation process generally involves the application of energy to convert the liquid formulation into droplets suitable for nasal or pulmonary delivery. Previous studies have shown that certain energy levels during aerosolisation can compromise drug stability by disrupting protective carriers (e.g., lipid nanoparticles) or altering the structural integrity of complex molecules (Van Rijn et al., 2023).

In this study, the impact of the aerosolisation process on DP stability was evaluated with an analytical toolkit: protein content was quantified using the BCA assay; complexation between the DS and the mucoadhesive excipient was assessed via Native-PAGE; particle size distribution was measured using DLS; and biological activity was evaluated through an indirect competitive ELISA. Across all analyses, the DP remained stable post-aerosolisation with only a minor increase in PDI and Z-average that might not necessarily be linked to changes in the DS (e.g., analytical and sample variability, interference from the device matrix). These data indicate that the BiVax device is capable of effectively aerosolising the DP without compromising its integrity.

An in-use stability study was also conducted to simulate potential exposure of the DP to the device under clinical conditions. During this study, both particle size and complexation with the mucoadhesive excipient remained stable. A slight decrease in protein concentration was observed after 4 h for the lower-concentration formulation and after 16 h for the higher-concentration formulation. After 16 h of incubation within the device, the biological activity had a slight reduction. Since the DP is not intended to be stored in the device before administration during clinical trials, these results actually suggest that transfer devices such as BiVax, where minimal contact between formulation and device is required, remain an interesting option for intranasal vaccination.

An initial assessment of the optimal administration configuration was conducted on a nasal cast model using an aqueous fluorescein solution. Across all tested configurations, deposition in the olfactory region remained negligible (below 3%), suggesting that intranasal administration of vaccines with physicochemical properties similar to water using BiVax is unlikely to pose neurological risks associated with direct central nervous system access via the olfactory pathway. This aspect was confirmed within a rabbit toxicity study, which demonstrated no damage to the olfactory bulb or neurological effects (data not presented). This finding is particularly relevant considering past safety concerns, such as those associated with the intranasal influenza vaccine Nasalflu (Berna Biotech), which was withdrawn from the market following reports of Bell’s palsy. The leading hypothesis for this adverse effect involved retrograde axonal transport of the *Escherichia coli* heat-labile enterotoxin adjuvant from the olfactory epithelium to the peripheral facial nerve (Lewis et al., 2009). It should also be noted that in the DP, no adjuvant has been added. Additionally, no detectable deposition was observed in the filter placed after the nasal cast (0%), indicating minimal to no exposure of the lower respiratory tract.

For immunisation via the nasal route to be effective, the vaccine must reach the lymphoid tissues. The nasopharynx, a key anatomical site housing the nasal-associated lymphoid tissue (NALT), remains a primary target for intranasal vaccine delivery (Cesta, 2006; Wilkins et al., 2021; Li et al., 2023). The nasal turbinates should also be considered as a priority target (Hejazi et al., 2026), either through the mucociliary clearance mechanism that will eventually transport the vaccine to the nasopharynx (Marttin et al., 1998; Laube et al., 2024), or through localised immune responses within the turbinates themselves via disseminated lymphoid tissue (Pabst, 2015; Gailleton et al., 2025).

Within this study, a combination of different administration configurations was assessed, including insertion angle (30, 45° and 50°) and depth (2, 9 and 15 mm), airflow (0 and 15 lpm) and nasal cast position (upright, 45° and supine). A multivariate analysis revealed that all parameters (insertion angle and depth, and nasal cast position), except airflow, significantly influenced regional deposition patterns. In a previous study conducted by Laube et al. on the same delivery device, the nasal cast position was identified as a determinant of deposition in the middle and lower turbinates, whereas insertion angle and depth were not found to be significant (Laube et al., 2024). These differences could be linked to the experimental design and the different nasal cavity anatomies. The prior study focused on different nasal cast models (adult and child) with nasal cast model positions (upright, 45° and supine), while evaluating a limited number of administration angles (30°–45°) and insertion depths (6 and 9 mm), and did not use a full factorial design of experiments, which could contribute to the lack of discriminatory ability of these variables. In contrast, the present study focused on a single nasal cast with a wider range of administration angle (30°–50° instead of 30°–45°) and insertion depth (2–15 mm instead of 6–9 mm) values, each assessed at three levels, thereby improving the resolution and discriminatory power of the analysis. The results indicate that a lower insertion angle (30°) combined with a greater insertion depth (15 mm) promotes enhanced deposition in the posterior nasal region, particularly within the lower turbinates. These findings are consistent with previous nasal cast studies by Hejazi et al. in both adult and pediatric models, where the researchers concluded that: non-extreme breathing patterns did not significantly affect drug delivery; administration angle and insertion depth have an impact on regional deposition; and not aiming upward is a key factor to enhance deposition in the turbinates, particularly in the lower turbinates (Hejazi et al., 2026). Similarly, studies by Kundoor and Dalby (2011), Warnken et al. (2018) demonstrated a larger deposition in the posterior region with a lower insertion angle and greater insertion depth, which is well aligned with computational fluid dynamics simulations by Akash et al. (2023) where an improved use actuation protocol, pointing to the back of the throat significantly increased the deposition in the targeted region (Williams and Suman, 2022). With an optimised angle and insertion depth, posterior deposition remained robust across all nasal cast orientations with the optimised angle and insertion depth, ranging from 82% to 94% on average, as observed by Laube et al. (2024).

The nasal cast model was also evaluated with the nasal vaccine formulation. The deposition results were similar to those with an aqueous fluorescein solution, indicating that the findings from the multifactorial analysis apply to this formulation. Deposition was predominantly localised in the posterior nasal region, with substantial coverage of the turbinates. This may be attributed to the broader spray angle and a droplet size distribution (DSD) optimized for nasal delivery, as previously reported elsewhere (Li et al., 2023; Hejazi et al., 2026). Minimal to no deposition was observed in the olfactory region and the filter. The latter finding is likely due to the low proportion of droplets smaller than 10 μm, as indicated by the DSD measurements.

Two *in vivo* preclinical studies were conducted to evaluate the administration of the DP. The initial study, performed in mice, aimed to determine the optimal time point for assessing fluorescein deposition in the nasal cavity. Results indicated that peak intranasal deposition occurred at 15 min post-administration, which was subsequently used as the reference time point in the rabbit study. A limitation of the rabbit *in vivo* study arises from the fact that the animals available to us belonged to a cohort originally allocated for regulatory toxicity assessments. Because those animals were earmarked for the mandatory toxicity program, which is not reported in this manuscript, we were restricted to testing only one animal per condition (BiVax vs. micropipette). Nevertheless, the intranasal administration of the formulation yielded a homogeneous distribution across both sides of the nasal cavity in the animal, suggesting a reliable and reproducible delivery procedure. This reliability was further corroborated by the highly consistent immunogenicity outcomes observed among the 20 animals allocated to each group (BiVax vs. micropipette) in the regulatory toxicity assay (data not shown). In the rabbit model, intranasal delivery using the BiVax spray device was compared to administration via micropipette and calculation by the total radiant efficiency measured within the regions of interest composed of: (1) nasal and maxillary turbinates, (2) ethmoid turbinates and (3) nasopharynx-oropharynx-trachea aera. The total deposition within the regions of interest following micropipette administration was lower than with the BiVax with a measured total radiant efficiency about 50% lower (5.10E+11 ± 2.14 vs. 2.50E+11 ± 8,23). Moreover, deposition with micropipette was poorly distributed, with an unbalanced distribution with a low localization of the formulation in the nasal and maxillary turbinates (11%) (Figure 10C). In contrast, BiVax administration resulted in broader and more uniform coverage across the nasal cavity. The unbalanced deposition with a high concentration of formulation in the ethmoid turbinates and pharynx aera observed with the micropipette may be attributed to the administration procedure, during which the rabbit was held in an inverted position during and for 1 minute following instillation.

The nasal cast and the *in vivo* experiment demonstrate the ability of the BiVax spray device to deliver the formulation extensively and homogeneously to key inductive sites of the mucosal immune response within the nasal cavity and the adenoid region, particularly the lower and intermediate turbinates and the nasopharynx nasal-associated lymphoid tissue (NALT).

## 5 Conclusion

This proof-of-concept study demonstrates the feasibility of delivering a protein-based intranasal formulation targeting SARS-CoV-2 using the Aptar Pharma BiVax spray device. The formulation, incorporating a mucoadhesive excipient, maintained its physicochemical integrity and biological activity following aerosolisation, as confirmed by multiple analytical techniques. In-use stability studies and the transitory character of BiVax further supported the robustness of the drug product under simulated clinical conditions.

Deposition studies using a nasal cast model revealed that optimised administration parameters (lower insertion angle and greater insertion depth) enhanced delivery to the posterior nasal region, including the turbinates and nasopharynx, while minimising deposition in the olfactory region and lower respiratory tract. These findings were confirmed with the vaccine formulation, indicating that the optimised delivery parameters could be considered when designing instructions for use for the final drug product.

*In vivo* preclinical studies in mice and rabbits confirmed the effective and homogeneous distribution of the formulation within the nasal cavity, particularly to regions associated with mucosal immune induction, such as the NALT. Compared to micropipette administration, the BiVax device achieved superior coverage and more physiologically relevant deposition patterns.

Collectively, these results support the use of the BiVax device for intranasal delivery of protein-based vaccines and highlight its potential to target key immunological sites while preserving formulation stability and safety. This study therefore confirmed the suitability of the device for the intranasal administration of the LVT-001 formulation in the context of clinical trial (ClinicalTrials.gov ID NCT06821126).

## Data Availability

The original contributions presented in the study are included in the article/supplementary material, further inquiries can be directed to the corresponding authors.

## References

[B1] Akash M. M. H. Lao Y. Balivada P. A. Ato P. Ka N. K. Mituniewicz A. (2023). On a model-based approach to improve intranasal spray targeting for respiratory viral infections. Front. Drug Deliv. 3, 1164671. 10.3389/fddev.2023.1164671 40838066 PMC12363323

[B2] Cesta M. F. (2006). Normal structure, function, and histology of mucosa-associated lymphoid tissue. Toxicol. Pathol. 34, 599–608. 10.1080/01926230600865531 17067945

[B3] Davis S. S. (2001). Nasal vaccines. Adv. Drug Deliv. Rev. 51, 21–42. 10.1016/S0169-409X(01)00162-4 11516777

[B4] Debertin A. S. Tschernig T. Tönjes H. Kleemann W. J. Tröger H. D. Pabst R. (2003). Nasal-associated lymphoid tissue (NALT): frequency and localization in young children. Clin. Exp. Immunol. 134, 503–507. 10.1111/j.1365-2249.2003.02311.x 14632758 PMC1808902

[B5] Durand M. Pourchez J. Louis B. Pouget J.-F. Isabey D. Coste A. (2011). Plastinated nasal model: a new concept of anatomically realistic cast. Rhinology 49, 30–36. 10.4193/Rhino09.187 21468371

[B6] Gailleton R. Mathew N. R. Reusch L. Schön K. Scharf L. Strömberg A. (2025). Ectopic germinal centers in the nasal turbinates contribute to B cell immunity to intranasal viral infection and vaccination. Proc. Natl. Acad. Sci. U. S. A. 122, e2421724122. 10.1073/pnas.2421724122 40112112 PMC11962485

[B7] Hejazi M. Owen X. Edwards D. J. Hindle M. Longest W. Schuman T. (2026). Regional nasal drug deposition in pediatric vs. adult models: *in vitro* insights into user technique and breathing patterns sensitivity. Pharm. Res. 43, 1141–1156. 10.1007/s11095-026-04066-8 41840267 PMC13092986

[B8] Kundoor V. Dalby R. N. (2011). Effect of formulation- and administration-related variables on deposition pattern of nasal spray pumps evaluated using a nasal cast. Pharm. Res. 28, 1895–1904. 10.1007/s11095-011-0417-6 21499839

[B9] Lakhrif Z. Poupée-Beaugé A. Boursin F. Ducournau C. Lantier L. Moiré N. (2025). Intranasal spike and nucleoprotein fusion protein-based vaccine provides cross-protection and reduced transmission against SARS-CoV-2 variants. NPJ Vaccines 10, 75. 10.1038/s41541-025-01123-y 40251181 PMC12008205

[B10] Laube B. L. Kesavan J. Farias G. Karavas N. Blondel M. Suman J. (2024). Targeting aerosol delivery to regions of nasal-associated lymphoid tissue (NALT) in three dimensional models of human intranasal airways using the BiVax intranasal atomizer. Front. Drug Deliv. 4, 1456538. 10.3389/fddev.2024.1456538 40836969 PMC12363273

[B11] Lewis D. J. M. Huo Z. Barnett S. Kromann I. Glemza R. Galiza E. (2009). Transient facial nerve paralysis (Bell’s palsy) following intranasal delivery of a genetically detoxified mutant of *Escherichia coli* heat labile toxin. PLoS One 4, e6999. 10.1371/journal.pone.0006999 19756141 PMC2737308

[B12] Li L. Wilkins J. V. Esmaeili A. R. Rahman N. Golshahi L. (2023). *In vitro* comparison of local nasal vaccine delivery and correlation with device spray performance. Pharm. Res. 40, 537–550. 10.1007/s11095-022-03452-2 36536098

[B13] Lobaina Mato Y. (2019). Nasal route for vaccine and drug delivery: features and current opportunities. Int. J. Pharm. 572, 118813. 10.1016/j.ijpharm.2019.118813 31678521

[B14] Madhavan M. Ritchie A. J. Aboagye J. Jenkin D. Provstgaad-Morys S. Tarbet I. (2022). Tolerability and immunogenicity of an intranasally-administered adenovirus-vectored COVID-19 vaccine: an open-label partially-randomised ascending dose phase I trial. EBioMedicine 85, 104298. 10.1016/j.ebiom.2022.104298 36229342 PMC9550199

[B15] Marttin E. Schipper N. G. M. Verhoef J. C. Merkus F. W. H. M. (1998). Nasal mucociliary clearance as a factor in nasal drug delivery. Adv. Drug Deliv. Rev. 29, 13–38. 10.1016/S0169-409X(97)00059-8 10837578

[B16] Pabst R. (2015). Mucosal vaccination by the intranasal route. Nose-associated lymphoid tissue (NALT)—Structure, function and species differences. Vaccine 33, 4406–4413. 10.1016/j.vaccine.2015.07.022 26196324

[B17] Schägger H. von Jagow G. (1991). Blue native electrophoresis for isolation of membrane protein complexes in enzymatically active form. Anal. Biochem. 199, 223–231. 10.1016/0003-2697(91)90094-A 1812789

[B18] Scherließ R. (2020). Nasal formulations for drug administration and characterization of nasal preparations in drug delivery. Ther. Deliv. 11, 183–191. 10.4155/tde-2019-0086 32046624

[B19] Southam D. S. Dolovich M. O’Byrne P. M. Inman M. D. (2002). Distribution of intranasal instillations in mice: effects of volume, time, body position, and anesthesia. Am. J. Physiology-Lung Cell. Mol. Physiology 282, L833–L839. 10.1152/ajplung.00173.2001 11880310

[B20] van Rijn C. J. M. Vlaming K. E. Bem R. A. Dekker R. J. Poortinga A. Breit T. (2023). Low energy nebulization preserves integrity of SARS-CoV-2 mRNA vaccines for respiratory delivery. Sci. Rep. 13, 8851. 10.1038/s41598-023-35872-4 37258559 PMC10231294

[B21] Warnken Z. N. Smyth H. D. C. Davis D. A. Weitman S. Kuhn J. G. Williams R. O. (2018). Personalized medicine in nasal delivery: the use of patient-specific administration parameters to improve nasal drug targeting using 3D-Printed nasal replica casts. Mol. Pharm. 15, 1392–1402. 10.1021/acs.molpharmaceut.7b00702 29485888

[B22] Wilkins J. V. Golshahi L. Rahman N. Li L. (2021). Evaluation of intranasal vaccine delivery using anatomical replicas of infant nasal airways. Pharm. Res. 38, 141–153. 10.1007/s11095-020-02976-9 33449250

[B23] Williams G. Suman J. D. (2022). *In vitro* anatomical models for nasal drug delivery. Pharmaceutics 14, 1353. 10.3390/pharmaceutics14071353 35890249 PMC9323574

[B24] Williams G. Cabrera M. Graine L. Le Guellec S. Hauchard N. Jamar F. (2021). *In vitro* and *in vivo* assessment of regional nasal deposition using scintigraphy from a nasal spray and a nasal powder. Respir. Drug Deliv. 135–140. Available online at: https://www.rddonline.com/rdd/article.php?id=0&sid=103&ArticleID=2785&return=1.

[B25] Wrapp D. Wang N. Corbett K. S. Goldsmith J. A. Hsieh C.-L. Abiona O. (2020). Cryo-EM structure of the 2019-nCoV spike in the prefusion conformation. Science. 367, 1260–1263. 10.1126/science.abb2507 32075877 PMC7164637

[B26] Yusuf H. Kett V. (2017). Current prospects and future challenges for nasal vaccine delivery. Hum. Vaccin. Immunother. 13, 34–45. 10.1080/21645515.2016.1239668 27936348 PMC5287317

